# Increasing Research Capacity in Underserved Communities: Formative and Summative Evaluation of the Mississippi Community Research Fellows Training Program (Cohort 1)

**DOI:** 10.3389/fpubh.2018.00021

**Published:** 2018-02-09

**Authors:** Danielle Fastring, Susan Mayfield-Johnson, Tanya Funchess, Candice Green, Victoria Walker, Georgette Powell

**Affiliations:** ^1^Department of Public Health, University of Southern Mississippi, Hattiesburg, MS, United States; ^2^Office of Health Disparity Elimination, Mississippi State Department of Health, Jackson, MS, United States; ^3^Office of Policy and Evaluation, Mississippi State Department of Health, Jackson, MS, United States

**Keywords:** community education, community-based participatory research, research capacity, health disparities, program evaluation, public health

## Abstract

**Background:**

The Mississippi Community Research Fellows Training Program (MSCRFTP) is a 15-week program conducted in Jackson, MS, USA consisting of training in the areas of evidence-based public health, research methods, research ethics, and cultural competency. The purpose of the program was to increase community knowledge and understanding of public health research, develop community-based projects that addressed health disparity in the participants’ community, increase individual and community capacity, and to engage community members as equal partners in the research process.

**Methods:**

A comprehensive evaluation of the MSCRFTP was conducted that included both quantitative and qualitative methods. All participants were asked to complete a baseline, midterm, and final assessment as part of their program requirements. Knowledge gained was assessed by comparing baseline assessment responses to final assessment responses related to 27 key content areas addressed in the training sessions. Assessments also collected participants’ attitudes toward participating in research within their communities, their perceived influence over community decisions, and their perceptions of community members’ involvement in research, satisfaction with the program, and the program’s impact on the participants’ daily practice and community work.

**Results:**

Twenty-one participants, the majority of which were female and African-American, completed the MSCRFTP. Knowledge of concepts addressed in 15 weekly training sessions improved significantly on 85.2% of 27 key areas evaluated (*p* < 0.05). Two mini-grant community based participatory research projects proposed by participants were funded through competitive application. Most participants agreed that by working together, the people in their community could influence decisions that affected the community. All participants rated their satisfaction with the overall program as “very high” (76.2%, *n* = 16) or “high” (23.8%, *n* = 5).

**Conclusion:**

The evaluation of the MSCRFTP demonstrates that participants have the necessary knowledge to engage as research partners, and the pilot projects provided an opportunity for application of this objective to be realized. Overall, the MSCRFTP was an intervention that assisted community members in identifying their communities’ strengths and weaknesses, interpret knowledge in a meaningful way, and create a self-reflective community of inquiry for change.

## Introduction

Disparities in socioeconomic levels, healthcare access and utilization, and education among communities and ethnic groups underscore the need to adjust service delivery and health education programs accordingly. The failure to secure optimal preventative care and treatment practices, and to achieve optimal self-care is rooted in numerous individual, environmental, and health care system-based variables. One individual factor that may lead to a reduction in seeking healthcare services is mistrust. Multiple studies have focused on medical research, negative encounters with health care personnel, and racial disparities in health. Gamble (1) describes the legacy of distrust between African-Americans and medical research (1). Additional studies highlight distrust of the medical community as a prominent barrier to participation in clinical research (2–5). Aspects of the built environment that may lead to health disparities may include poor neighborhood walkability, a lack of safe spaces to play or exercise (6, 7), or food insecurity (8, 9). The ability to successfully navigate the ever-changing health care system can also predict overall health status (10).

Even after taking these factors into consideration, many interventions continue to lack the ability to reduce health disparities and improve health outcomes. Considering an individual’s community is a powerful force in his or her lives, standard individual-based health interventions may not be suitable for long-lasting change. Innovation in developing or refining interventions to include broader community-based dimensions can improve outcomes.

Community-based participatory research (CBPR) is one such strategy to address these needs and is defined as a “collaborative approach to research that equitably involves all partners in the research process and recognizes the unique strengths that each brings” (11). The CBPR approach has been found to be effective in prioritizing and implementing health promotion projects that impact health and health disparities (12–14). Developing interventions to solve community problems can occur through social engineering, new knowledge production, and transformational leadership inspired to create a self-reflective community of inquiry (11).

Through this participatory process, information is exchanged freely, and all partners share problem-solving to accomplish knowledge attainment. The community is a unit of identity with existing strengths and resources upon which to build this process. Additionally, the resources and expertise of research partners are employed to benefit all stakeholders. CBPR focuses on local public health problems and ecology while recognizing that there are multiple determinants of health (11).

Community-based participatory research has been shown to be effective in facilitating the establishment of academic-community partnerships (15, 16). When utilizing a CBPR framework in creating this type of partnership, it is imperative that the partnership is equitable with regards to research responsibilities, and that members from each side of the partnership are involved in all aspects of the research process (17–19). This equity can be compromised when community members lack the power that comes from having knowledge based in the foundations of general public health and basic research methods.

In an effort to improve mistrust between racial and ethnic communities and research, foster academic-community partnerships, and to build individual and community research capacity through the education of community stakeholders in Jackson, Mississippi, the Mississippi State Department of Health, Office of Health Disparity Elimination, adapted the Community Research Fellows Training Program (CRFT) of the Division of Public Health Sciences at Washington University School of Medicine and the Siteman Cancer Center (SCC) in St. Louis, Missouri (20–22). The Mississippi Community Research Fellows Training Program was the result of that adaptation. The purpose of this article is to present findings from a comprehensive evaluation that was conducted with the first enrolled cohort of the program.

## Materials and Methods

### Mississippi Community Research Fellows Training Program (MSCRFTP)

The MSCRFTP is a community health training course designed to equip community members with an understanding of public health, factors that influence protecting and improving the health of people and their communities, research methods and skills, and a fundamental understanding of research funding. The MSCRFTP’s purpose was to promote the role of racial/ethnic minorities and other underserved populations in public health research through CBPR and community engagement to meet the following objectives:

1. Increase community knowledge and understanding of public health research,
2. Develop CBPR projects that address community-identified health disparity projects in the greater Jackson metro-area,
3. Increase individual and community capacity, and
4. Enable community members with leadership and skill development to engage as equal partners in research processes (21).

Prior to program delivery and to inform adaptation from the original CRFT program, both a steering committee and a community advisory board (CAB) were established. Participants in these two groups were drawn from the Mississippi Department of Health, the five academic institutions in the region, and several community-based organizations. The academic institutions included Jackson State University, Mississippi State University, Tougaloo College, University of Mississippi Medical Center, and the University of Southern Mississippi. Community organizations consisted of Building Bridges, Foundation for the Mid South, My Brother’s Keeper, Inc., and the Partnership for a Healthy Mississippi. With guidance from the steering committee and the CAB, the MSCRFTP was culturally adapted from the original CRFT program that was piloted by the Program to Eliminate Cancer Disparities at the SCC, Barnes Jewish Hospital, and the Division of Public Health Sciences at Washington University School of Medicine (20–22). The original CRFT program is adapted from the Community Alliance for Research Empowering Social change (CARES) Fellows Research Training, which was designed to implement culturally appropriate ways to increase scientific literacy among community members (23, 24). The 19 topic areas in the original CRFT curriculum were retained for MSCRFTP; however, session content was adapted to ensure relevance to health disparities in Mississippi and areas of social inequity.

Participants in the first MSCRFTP cohort were recruited using culturally competent advertisements and recruitment information sessions in Jackson, Yazoo, and Simpson Counties of Mississippi. Participants had to submit an application and letters of recommendation for course selection, and agree to attend an introduction session, 15 weekly courses, and upon completion of the MSCRFTP, a graduation ceremony. Participants also completed a baseline assessment, mid-point assessment, and final assessment in addition to weekly pre-and post-tests for each topic and several out of class homework assignments.

The MSCRFTP covered 19 topic areas during weekly 3 h classes held for 15-consecutive weeks. The session topics and learning objectives can be found in Table S1 in Supplementary Material. A similar list from the original CRFT program can be found in D’agostino-McGowan (21). Sessions were held from August 26, 2014 to December 9, 2014. Each session was taught by faculty recruited by the Director of the MS Department of Health, Office of Health Disparity Elimination. Faculty consisted of community health professionals, subject matter experts, and faculty from the five academic institutions previously mentioned. Most topics mirrored those offered in the original CRFT program. Topics aligned with curriculum requirements of a Master of Public Health program, but were condensed and delivered in such a way that community members would find the information accessible and applicable to their community experience.

### Comprehensive Evaluation

A comprehensive evaluation of the MSCRFTP was conducted that included both quantitative and qualitative methods. All participants were asked to complete a baseline, midterm, and final assessment as part of their program requirements. All assessments were administered *via* Qualtrics (Qualtrics, Provo, UT, USA). The baseline assessment was administered prior to the first training session, and included items that assessed the participants’ knowledge about 27 key concepts that would be covered in the future training sessions. The baseline assessment also included items that captured the participants’ attitudes toward participating in research within their communities, their perceived influence over community decisions, and perceptions of community members’ involvement in research. The mid-point assessment, administered between the 7th and 10th week of the training program, consisted of items that assessed participant satisfaction with topics presented, open-ended questions to illicit strengths and weaknesses of the program, and participants’ preferred content delivery method. Participants were also asked to provide examples of how the MSCRFTP had impacted their daily practice and community work. The final assessment was administered after the training modules were completed. The final assessment included the same knowledge items that were included in the baseline assessment so that knowledge gains could be assessed. Additionally, participants were asked to report the topics that they found most useful, faculty members that enhanced learning, and the sessions that were most enjoyable. Last, the final assessment included items related to participants’ satisfaction with the MSCRFTP overall. A logic model was created which described program inputs, activities, outputs, and short-term, intermediate, and long-term outcomes (Figure S1 in Supplementary Material).

## Results

### Participant Demographics

Twenty-seven participants completed the baseline assessment. Demographic characteristics of the first MSCRFTP cohort can be found in Table 1. Most of the group completing the baseline assessment were female (*n* = 23, 85.2%) and African-American (*n* = 23, 85.2%). The remaining participants reported their race as Caucasian (*n* = 3, 11.1%), or other (*n* = 1, 3.7%). All participants reported an ethnicity of Non-Hispanic (*n* = 27, 100.0%). Almost all participants were born in the United States (*n* = 26, 96.3%) with one participant’s birthplace listed as Canada. Most participants lived in Jackson, MS (*n* = 12, 44.4%). Others reported living in cities that surrounded the capital of Mississippi, such as Brandon (*n* = 4, 14.8%), Clinton (*n* = 3, 11.1%), Madison (*n* = 2, 7.4%), Piney Woods (*n* = 1, 3.7%), Port Gibson (*n* = 1, 3.7%), Ridgeland (*n* = 2, 7.4%), and Vicksburg (*n* = 2, 7.4%).

**Table 1:** Demographic characteristics of community research fellows training program at baseline (*n* = 27).

Characteristic	*n*(%)
**Gender**	
Female	23 (85.2)
**Race**	
African-American	23 (85.2)
Caucasian	3 (11.1)
Other	1 (3.7)
**Ethnicity**	
Non-Hispanic	27 (100)
**Country of origin**	
United States	26 (96.3)
Canada	1 (3.7)
**City of residence in Mississippi**	
Brandon	4 (14.8)
Clinton	3 (11.1)
Jackson	12 (44.4)
Madison	2 (7.4)
Piney Woods	1 (3.7)
Port Gibson	1 (3.7)
Ridgeland	2 (7.4)
Vicksburg	2 (7.4)
**Highest level of education**	
Some college or associates degree	3 (11.1)
College degree	8 (29.6)
Graduate degree	16 (59.3)
**Number of research classes completed**	
5 or more	2 (7.4)
3–4	4 (14.8)
1–2	11 (40.7)
None	10 (37.0)
**Current employment status**	
Full time	21 (77.8)
Part time	1 (3.7)
Unemployed	5 (18.5)

Participants ranged from 25 to 65 years of age ($\bar{X}$ = 44.3 years, ±13.5 years). All participants had attended college, with approximately one-third (*n* = 8, 29.6%) completing a college degree. More than half (*n* = 16, 59.3%) had completed graduate degrees. The participants’ experience with regards to research classes varied. More than one-third (*n* = 10, 37.0%) had never taken a research class prior to participation in the fellowship training program. Some reported that they had taken 1–2 research classes (*n* = 11, 40.7%), a few reported that they had taken 3–4 research classes (*n* = 4, 14.8%), and the remaining participants (*n* = 2, 7.4%) reported taking five or more research classes. The majority of participants worked full time (*n* = 21, 77.8%), only one participant (3.7%) worked part time, and five participants (18.5%) were unemployed. Additionally, 22.2% (*n* = 6) of participants were students, 7.4% (*n* = 2) were retired.

### Perceived Influence over Decisions Impacting Communities

Most participants (*n* = 25, 92.6%) either “agreed” or “strongly agreed” that by working together, the people in their community could influence decisions that affected the community (Table 2). However, only approximately half (*n* = 13, 48.15%) “agreed” or “strongly agreed” that people in their community could work together to influence decisions at a local, state, or national level. Fewer still (*n* = 7, 25.93%) were satisfied with the amount of influence that they, themselves, had on decisions that affected their community.

**Table 2:** Perceived influence over decisions impacting the MSCRFT participants’ communities.

Question	Strongly disagree*n*(%)	Disagree*n*(%)	Neutral*n*(%)	Agree*n*(%)	Strongly agree*n*(%)
By working together, people in my community can influence decisions that affect the community	0 (0.00)	2 (7.41)	0 (0.00)	7 (25.93)	18 (66.67)
People in my community work together to influence decisions at a local, state, or national level that affect the community	2 (7.41)	4 (14.81)	8 (29.63)	7 (5.93)	6 (22.22)
I am satisfied with the amount of influence that I have on decisions that affect my community	5 (18.52)	6 (22.22)	9 (33.33)	5 (18.52)	2 (7.41)

### Community Involvement in Research

Of the 16 specific aspects of research analyzed (Table 3), more than half of the participants thought that community members should be “quite a bit involved” or “extremely involved” in defining the research problem (85.16%), deciding on issues to research (74.08%), and recruiting study participants (62.97%). There were a few aspects of research that were consistently rated by participants as areas with which community members should not be involved. These areas were interpreting study findings (48.15%), analyzing collected data (40.74%), writing reports and journal articles (40.74%), choosing research methods (29.63%), and developing sampling procedures (29.63%).

**Table 3:** Participants’ view of how involved members of the community should be during specific aspects of the research process (*n* = 27).

Aspect of research	Not at all involved*n*(%)	A little bit involved*n*(%)	Somewhat involved*n*(%)	Quite a bit involved*n*(%)	Extremely involved*n*(%)
Defining the problem	0 (0.00)	2 (7.41)	2 (7.41)	7 (25.93)	16 (59.26)
Deciding on issues to research	0 (0.00)	1 (3.70)	6 (22.22)	10 (37.04)	10 (37.04)
Developing research questions	1 (3.70)	2 (7.41)	15 (55.56)	5 (18.52)	4 (14.81)
Designing interview and/or survey questions	1 (3.70)	5 (18.52)	13 (48.15)	5 (18.52)	3 (11.11)
Collecting data	3 (11.11)	7 (25.93)	7 (25.93)	5 (18.52)	5 (18.52)
Recruiting study participants	3 (11.11)	1 (3.70)	6 (22.22)	10 (37.04)	7 (25.93)
Analyzing collected data	11 (40.74)	3 (11.11)	7 (25.93)	2 (7.41)	4 (14.81)
Disseminating and sharing findings	2 (7.41)	6 (22.22)	5 (18.52)	5 (18.52)	9 (33.33)
Grant proposal writing	6 (22.22)	7 (25.93)	5 (18.52)	7 (25.93)	2 (7.41)
Choosing research methods	8 (29.63)	10 (37.04)	5 (18.52)	1 (3.70)	3 (11.11)
Developing sampling procedures	8 (29.63)	7 (25.93)	7 (25.93)	2 (7.41)	3 (11.11)
Implementing the intervention	3 (11.11)	5 (18.52)	5 (18.52)	8 (29.63)	6 (22.22)
Collecting primary data	6 (22.22)	9 (33.33)	5 (18.52)	2 (7.41)	5 (18.52)
Interpreting study findings	13 (48.15)	6 (22.22)	3 (11.11)	3 (11.11)	2 (7.41)
Writing reports and journal articles	11 (40.74)	10 (37.04)	4 (14.81)	0 (0.00)	2 (7.41)
Giving presentations at meetings and conferences	2 (7.41)	3 (11.11)	12 (44.44)	7 (25.93)	3 (11.11)

### Formative Evaluation

The formative evaluation phase of the MSCRFTP included asking questions pertaining to topics covered in the initial weeks of the training program, the development of a logic model (Figure S1 in Supplementary Material), identifying strengths and weaknesses of the program, and participants’ preferred education methods in the early stages of program implementation.

To learn about participants’ opinions about the MSCRFTP, a mid-point assessment was administered. Twenty-four participants completed the assessment. Open-ended questions allowed for a qualitative and thorough assessment of participants’ opinions to be garnered while allowing time to implement suggested changes during the remainder of the program. Most participants (*n* = 23, 95.83%) “agreed” or “strongly agreed” that the MSCRFTP staff was knowledgeable and helpful. Similarly, 95.8% (*n* = 23) would recommend the MSCRFTP to others in their community.

Mississippi Community Research Fellows Training Program participants were asked to list the three topics that they felt were most important to them during the first 9 weeks of the training program. The most often selected topics were *Health Disparities* (*n* = 9, 37.50%), *Logic Models* (*n* = 7, 29.17%), and *Conducting Community Research* (*n* = 6, 25.00%). When asked about topics that they would like to learn more about, approximately half (*n* = 11, 45.83%) answered that there were none. Participants (*n* = 3, 12.50%) cited topics that were planned to be taught in sessions 10–15 (i.e., grant writing). Topics that were not within the scope of the MSCRFTP were topics related to statistical analysis, disparities specific to Mississippi, mental health and youth, violence, climate change, drug abuse, clinical tools associated with community health research, program implementation, goal setting, and the overall public health framework.

### Program Strengths and Weaknesses

To assess program strengths and weaknesses, participants were asked to list the three greatest strengths and weaknesses of the training. They overwhelmingly discussed the quality and nature of the program. The most frequently reported strength (*n* = 19, 79.17%) was related to the faculty presenters who taught the course modules. Characteristics of faculty presenters that were specifically listed were knowledge, diversity, accessibility, preparedness, quality, engagement with students, and presentation skills. The next most frequently cited strength (*n* = 8, 33.33%) was related to the MSCRFTP staff. They were commended as being detailed, reliable, friendly, and qualified. Variation of teaching methods was reported as strength by 25.00% (*n* = 6) of participants. Specific methods cited were quiz bowl, application activities, question and answer sessions, lectures and presentations, and pre- and post-tests.

When asked to comment on program weaknesses, approximately half (*n* = 11, 45.83%) reported that there were none. The most frequently cited weaknesses were related to the logistics of training (*n* = 7, 29.17%). Specifically, participants noted classroom distractions, such as sidebar conversations, frequency of breaks, days of the week that training was offered, and small print on handouts. Not having enough time was cited by 20.83% (*n* = 5) of participants. Four participants (16.67%) reported that there was inconsistency among presenters. Other comments were only cited by one or two participants and included time spent taking pictures every week (with the faculty presenters), and the length of the program, or food choices provided as part of the training.

Though 27 participants were originally accepted into the program, 21 completed the program in its entirety. Thus, the program completion rate was 77.8%. Reasons provided for leaving the program prior to completion were largely related to the time commitment required by the program.

Assessment of program strengths and weaknesses also lend to the success of the MSCRFTP in establishing skill development and building capacity. The program’s impact on community capacity was evaluated through open-ended qualitative survey questions. Participants gained skills in community assessment, grant writing, human subject’s protocols, and research methods. Strengths of the program cited were the quality and nature of the program, diverse and knowledgeable faculty presenters, strength of the MSCRFTP staff, and methodology presented in the program, such as the outside assignments, application activities, and question and answer sessions. Application to daily practice and community work was noted among the participants. One participant provided this example,

First and foremost, how I interact with the individuals and groups when I speak (cultural competency), the way in which I receive and process data (quantitative and qualitative), take more pictures to show and tell my community and anyone I am speaking the point I am trying to convey (photovoice), and continue to do my part as a member of the community to provide awareness and education in an effort to promote and maintain public health (community health and community-based prevention). I gained a wealth (policy research, grant writing, etc.) of information that will aid me on my present and future endeavor.

Another participant commented, “I will utilize the information obtained in the photovoice activity, playground assessment, community assessment to recommend community changes (tearing down abandoned houses, obtaining security for playgrounds, looking for grant money to establish a farmer’s market). I plan to work with neighborhood associations, city officials, and the District 28 Senator to implement these changes.”

Improvement of skills and networking were also noted as examples of how the training impacted practice and community work. One participant noted that “the cultural competency (session) will help me deal with the Hispanic population better.” Another participant clarified MSCRFTP as a case for skill enhancement, “I feel that I will be much better at analyzing the strengths, weaknesses, and possibilities of the communities and organizations I work with to contribute to the discussion on how to improve them.” Opportunities for networking include “I have made connections with people” and “networking with others” as beneficial to community engagement and enlarging the capacity of communities to address issues.

#### Learning Styles and Impact on Participants

When participants were asked to characterize the type of education method that they preferred, the most frequently cited method was group exercises (*n* = 16, 66.67%). This method was followed by case studies (*n* = 14, 58.33%) and lectures (*n* = 13, 54.17%). When asked to provide an example of how the MSCRFTP impacted their lives, the most frequently provided answer was that the program had impacted their daily practice and community work (*n* = 9, 37.50%). Comments received from participants include the following:

I now ask, ‘What questions do you have?’ instead of ‘Do you have any questions?’

If I am working in the clinic, I attempt to explain things in a simple manner.

I watch more health-related news and I read more health-related news. Also, prior to the training, I always thought of community work and change being accomplished through an employment site. I now embrace completely the concept of individuals as change agents.

Approximately one-third (*n* = 8, 33.33) reported changes in knowledge as the greatest impact of the program. Comments received included:

I can assess the resources, potential, and weaknesses of communities now.

This training has improved my daily practice by helping me to understand certain terminology that I was not familiar with.

Impacts related to service and skills improvement were reported by 12.50% (*n* = 3) of participants.

One example of service impacts included, “The training is helping me think more about how I can be an asset in my community in terms of promoting better health outcomes for a better quality of life.” One participant illustrated this as an example of skill enhancement, “I have to present 4 out of 5 days a week. This training has taught me to be effective and efficient by teaching me to go to the right websites, which has saved me so much time in research. I needed this class to help me with conference presentations when presenting posters at conferences.”

### Summative Evaluation

The purpose of the summative evaluation was to determine if significant knowledge gains were made by program participants, identification of sessions that were the most well-received by participants, and to determine the program’s impact on community capacity among underserved minorities to become more involved in CBPR. The program’s impact on knowledge gains was assessed by comparing the quality of answers provided at the baseline and final assessments related to key terms and concepts that were considered essential to the MSCRFTP training (Table 4). Baseline and final assessment data was linked *via* MSCRFTP participant identification number. Data were entered into Qualtrics (Qualtrics, Provo, UT, USA) and the quality of the answers was coded as follows based on a rubric:

0: the respondent reported that they did not know the answer, or did not provide an answer,
1: the respondent provided an answer, but it was incorrect,
2: the respondent provided an answer that contained two to three keywords, and was somewhat familiar with the concept or definition,
3: the respondent provided an answer and demonstrated a clear understanding of the concept or definition.

**Table 4:** Knowledge gained by MSCRFT participants baseline vs. final assessment.

	Score increased*n*(%)	% of MSCFRT fellows demonstrating clear understanding of concept			McNemar’s test		
			
		Baseline*n*(%)	Final*n*(%)	Test statistic	*p*-Value
What is informed consent?	21 (100.0)	3 (14.2)	16 (76.2)	11.1	<0.001
What is the Belmont Report?	17 (81.0)	1 (4.8)	12 (57.1)	9.1	0.003
What is the Tuskegee experiment?	16 (76.2)	5 (23.8)	20 (95.2)	13.1	<0.001
Define health literacy	16 (76.2)	3 (11.1)	18 (85.7)	13.1	<0.001
Define evidenced based public health	14 (66.7)	1 (4.8)	13 (61.9)	10.1	0.002
Define cultural competency	17 (81.0)	0 (0.0)	13 (61.9)	11.1	<0.001
What role does the IRB play in research?	14 (66.7)	3 (14.3)	14 (66.7)	7.7	0.003
What is HIPAA?	5 (23.4)	3 (14.3)	4 (19.0)	0.3	0.617
Explain the difference between qualitative and quantitative research methods	18 (85.7)	3 (14.3)	19 (90.5)	12.5	<0.001
What is the difference between primary and secondary data?	20 (95.2)	1 (4.8)	20 (95.2)	17.1	<0.001
Explain the difference between community-based participatory research and traditional research	17 (81.0)	1 (4.8)	8 (38.1)	4.0	0.020
What is epidemiology?	18 (85.7)	4 (19.0)	21 (100.0)	15.1	<0.001
What is a clinical trial?	21 (100.0)	0 (0.0)	21 (100.0)	19.0	<0.001
What is the mixed methods approach?	16 (76.2)	4 (19.0)	11 (52.4)	4.0	0.020
What is photovoice?	21 (100.0)	0 (0.0)	21 (100.0)	19.1	<0.001
What is the purpose of a focus group?	20 (95.2)	1 (4.8)	20 (95.2)	18.1	<0.001
What type of information should you expect to get from a community health assessment?	7 (33.3)	0 (0.0)	1 (4.8)	0.0	1.000
Describe the health promotion planning model that you believe is best to prevent and reduce substance abuse in an African-American community	7 (33.3)	4 (19.0)	1 (4.8)	1.3	0.124
What are the social determinants of health?	13 (61.9)	2 (9.5)	9 (42.9)	4.0	0.020
List three social determinants of health?	17 (81.0)	4 (19.0)	21(100.0)	15.1	<0.001
What is research?	19 (90.5)	0 (0.0)	14 (66.7)	12.1	<0.001
Define racial health disparities	14 (66.7)	2 (9.5)	9 (42.9)	4.0	0.020
What are the components of a SMART goal?	14 (66.7)	6 (28.6)	17 (81.0)	9.1	0.001
What is an odds ratio?	13 (61.9)	2 (9.5)	0 (0.0)	0.5	0.240
What is a *p* value?	17 (81.0)	0 (0.0)	9 (42.9)	7.1	0.004
List an effective method to advocate for a specific health issue in your community	16 (76.2)	1 (4.8)	8 (38.1)	5.1	0.012
How is research used to develop health policy?	15 (71.4)	0 (0.0)	8 (38.1)	6.1	0.007

Between the baseline and final assessment, participants significantly increased their knowledge score, as evidenced by a significant McNemar’s test statistic (*p* < 0.05) on 23 of 27 questions (85.19%) (Table 4). The questions with the biggest gains were items asking, “What is photovoice?” and “What is a clinical trial?” Both of these questions improved from no correct answers given at baseline to 100% of answers correct at the final assessment. Questions without significant improvement were, “What is HIPAA?” (*p* = 0.3) and “What type of information should you expect to get from a community needs assessment?” (*p* = 1.0). There were two items that had lower scores at the final assessment than the baseline assessment. These items were, “Describe the health promotion planning model that you believe is the best to prevent and reduce substance abuse in an African-American community?” (*p* = 0.12) and “What is an odds ratio?” (*p* = 0.24).

### Participant Feedback

#### Topics Enjoyed Most by Participants

During the final assessment, participants (*n* = 21) were asked to identify the three training sessions they enjoyed the most throughout the program. Participants reported on a variety of sessions and specific speakers. The most enjoyed sessions were Session IV: cultural competency (*n* = 8, 38.10%), Session XI: qualitative methods (*n* = 7, 33.33%), Session II: research methods (*n* = 6, 28.57%), and Session III: health disparities (*n* = 6, 28.57%).

#### Opinions about Training Course Overall

Participants were asked to rate their agreement with statements (Table 5) related to the training course overall. The majority of participants responded favorably to all statements reflecting a positive experience overall. When asked, 100% (*n* = 21) of respondents reported that they would recommend the Community Research Fellows Training Program to others. Respondents were also asked to provide an overall rating for the community research fellows training. Most, 76.2% (*n* = 16) rated the program “very high”, and the remaining participants, 23.8% (*n* = 5) rated the program as “high.”

**Table 5:** Participants’ level of agreement with statements regarding the Mississippi community research fellows training program (*n* = 21).

	Strongly disagree*n*(%)	Disagree*n*(%)	Neutral*n*(%)	Agree*n*(%)	Strongly agree*n*(%)
An appropriate amount of material was covered during this training	0 (0.00)	1 (4.76)	0 (0.00)	4 (19.05)	16 (76.19)
The facilitator(s) have been prepared and well organized	0 (0.00)	0 (0.00)	0 (0.00)	6 (28.57)	15 (71.43)
The facilitator(s) seemed knowledgeable about this subject	0 (0.00)	0 (0.00)	0 (0.00)	4 (19.05)	17 (80.95)
The information learned in this training was helpful	0 (0.00)	0 (0.00)	0 (0.00)	5 (23.81)	16 (76.19)
The structure and format of the training was beneficial to the learning process	0 (0.00)	0 (0.00)	0 (0.00)	6 (28.57)	15 (71.43)
The training location was convenient for me	0 (0.00)	0 (0.00)	0 (0.00)	9 (42.86)	12 (57.14)
The timing of the training sessions fit into my schedule	0 (0.00)	0 (0.00)	1 (4.76)	5 (23.81)	15 (71.43)
I was satisfied with the training facilities (classrooms, meeting spaces, furniture, parking, etc.)	0 (0.00)	0 (0.00)	1 (4.76)	4 (19.05)	16 (76.19)
Homework assignments were useful	0 (0.00)	0 (0.00)	0 (0.00)	5 (23.81)	16 (76.19)
The amount of homework was appropriate	0 (0.00)	0 (0.00)	0 (0.00)	6 (28.57)	15 (71.43)
Homework assignments helped me to better understand the lecture material presented to me	0 (0.00)	0 (0.00)	0 (0.00)	4 (19.05)	17 (80.95)
Small group activities and discussions were helpful and beneficial to my learning	0 (0.00)	0 (0.00)	3 (14.29)	5 (23.81)	13 (61.90)

## Discussion

Through formative and summative evaluation processes, it was determined that participants’ knowledge gain and satisfaction with the MSCRFTP were consistent to evaluations from the original CRFT training program (20, 21). Findings suggest that participants did increase their knowledge about public health research as evident through their significant increase in scores from baseline assessment to the final assessment.

Qualitative comments like “I am more aware of health and health issues,” “I am more mindful of being healthy and informing the people around me,” and “It MSCRFTP rounded out my daily practice by making me more aware of the health literacy of the population I am involved with” exemplify changes in knowledge that enhance community practice. The two questions without significant improvement may not necessarily reflect an absence of change in knowledge, but rather difficulty in wording of the question to the assessment of answers provided by participants. The question, “what is HIPAA” requires that the acronym be spelled out with an explanation of HIPAA. As a result, formative evaluation may require re-wording of the question or more specific instructions to answer the question.

Participants were given the opportunity to continue into the research phase of the training program, whereby they would write a proposal to compete in a $1,000 mini-grant competition to implement a project focused on improving the health of their community. All but two participants stated that they would like to continue on to the research phase of the MSCRFTP project (90.5%, *n* = 19). Many respondents (71.4%, *n* = 15) reported that they planned to submit a proposal to the MSCRFTP research phase project.

The evaluation utilized a mixed methods approach to conduct the formative and summative evaluation, which included the collection of quantitative and qualitative data. The qualitative data was beneficial in providing context to the quantitative data and will be utilized to inform programmatic changes for future MSCRFTP cohorts. Practical recommendations include providing an opportunity for participants to provide feedback at every session and through an anonymous process. Issues raised relating to classroom conditions, such as temperature, lighting, acoustics, and other concerns should be addressed quickly where possible to maintain an environment conducive to learning and active participation. Care should be taken to involve appropriate community partners who can contribute knowledge with regard to the needs and resources available in the community. Additionally, it is important to recruit faculty to the program that have experience in community-based interventions.

It is important to note that the number of participants who provided both pre- and post-data in this cohort was 21; thus, the findings may not be generalizable to the broader population. Findings from this cohort will be utilized to improve delivery of the intervention to subsequent cohorts of Mississippi research fellows. Comprehensive evaluation of future cohorts is needed to further substantiate this program’s effectiveness.

## Conclusion

The MSCRFTP was successful in enhancing participant knowledge and skill development in public health research topics. Overall, 21 participants completed the first training cohort of the MSCRFTP with significant increases in knowledge and application of research and skill development. At the initiation of the program, MSCRFTP set out to achieve four objectives.

The first objective was to increase community knowledge and understanding of public health research. From the quantitative analysis, there is evidence that there was a significant improvement in knowledge from the baseline assessment to the final assessment on 23 of 27 key concept questions. The mid-point assessment assisted in evaluating learning objectives, appropriateness of course content and delivery, and relevance of topic sessions to participants.

The second objective was to develop CBPR projects that addressed community-identified health disparity projects in the greater Jackson metro-area. The knowledge and skills gained, as demonstrated through the results, was a catalyst for the development of pilot projects supported through mini-grants to the Mississippi State Department of Health, Office of Health Disparity Elimination.

MSCRF’s created groups of cohort members based on their research interests and proposed projects for funding through mini-grants. Two group projects were funded. Group one implemented a health and wellness education project in an underserved area in Jackson, MS, USA which provided 6 months of classes pertaining to nutrition, exercise, and successfully managing chronic illnesses. The group partnered with a local Federally Qualified Health Center (FQHC) that offered services to the community members participating in the educational program. The FQHC calculated participants’ body mass index (BMI) and provided blood pressure and diabetes screenings.

The second group’s proposal involved educating the Parent and Teacher Association in Hinds County about the importance of implementing sex education classes in the school setting. Currently, Mississippi’s policy regarding school-based sex education requires parents to opt their child into participating in the class. This requirement leads to low student participation in the class, as many parents are unaware that the class is offered.The group’s primary goals were to increase parental awareness of the classes and to work with policy advocates to introduce a more effective bill during the upcoming legislative session.

The third objective of the MSCRFTP was to increase individual and community capacity. Participant comments, pilot project applications, and funding of the two pilot projects demonstrate the development of additional capacity among participants, and with other community members and organizations. The pilot projects are currently ongoing in the greater Jackson metropolitan area.

Finally, the fourth objective was to enable community members with leadership and skill development to engage as equal partners in research processes. MSCRFTP was a community training for members of the community to understand what public health is, the factors that influence public health, research methodology, and grantsmanship. Topics aligned closely with the curriculum requirements for a Masters of Public Health degree, but were condensed and delivered in appropriate messages and methods to ensure that community members would find the information accessible and applicable. Topics covered the spectrum of evidence-based public health from the research process, to research methods, both quantitative and qualitative analysis, to research ethics, synthesis, and evaluation. The evaluation presented here demonstrates that participants have the necessary knowledge to engage as research partners, and the pilot projects provided an opportunity for application of this objective to be realized. Overall, the MSCRFTP was an intervention that assisted to help community members identify their community strengths and weaknesses, interpret knowledge in a meaningful way, and create a self-reflective community of inquiry for change.

The MSCRFTP has since been repeated with a second and third cohort of research fellows with similar findings (manuscripts in preparation). A fourth Mississippi cohort is in the planning stages. The original CRFT curriculum is adaptable and is recommended to be utilized with virtually any community to achieve the above objectives (22).

## Ethics Statement

This study was carried out in accordance with the recommendations of Federal Drug Administration regulations (21 CFR 26, 111), Department of Health and Human Services (45 CFR Part 46), and University of Southern Mississippi guidelines of the Institutional Review Board with written informed consent from all subjects. All subjects gave written informed consent in accordance with the Declaration of Helsinki. The protocol was approved by the USM Institutional Review Board (Project Number: 16042501).

## Author Contributions

DF provided quantitative data analysis, SM-J provided qualitative data analysis, DF, SM-J, TF, CG, VW, and GP participated in drafting and editing the article for publication. All authors contributed to manuscript revision, read, and approved the submitted version.

## Conflict of Interest Statement

The authors declare that the research was conducted in the absence of any commercial or financial relationships that could be construed as a potential conflict of interest.

## References

[1] Gamble VN A legacy of distrust: African Americans and medical research. Am J Prev Med (1993) 9(6 Suppl):35–8. 8123285

[2] Cain GE Kalu N Kwagyan J Marshall VJ Ewing AT Bland WP Beliefs and preferences for medical research among African-Americans. J Racial Ethn Health Disparities (2016) 3(1):74–82. 10.1007/s40615-015-0117-8 26896107 PMC5177967

[3] Corbie-Smith G Thomas SB St George DMM. Distrust, race, and research. Arch Intern Med (2002) 162(21):2458–63. 10.1001/archinte.162.21.2458 12437405

[4] Corbie-Smith G Thomas SB Williams MV Moody-Ayers S. Attitudes and beliefs of African Americans toward participation in medical research. J Gen Intern Med (1999) 14(9):537–46. 10.1046/j.1525-1497.1999.07048.x 10491242 PMC1496744

[5] Kennedy BR Mathis CC Woods AK. African Americans and their distrust of the health care system: healthcare for diverse populations. J Cult Divers (2007) 14(2):56–60. 19175244

[6] Gelormino E Melis G Marietta C Costa G. From built environment to health inequalities: an explanatory framework based on evidence. Prev Med Rep (2015) 2:737–45. 10.1016/j.pmedr.2015.08.019 26844145 PMC4721462

[7] Smith M Hosking J Woodward A Witten K MacMillan A Field A Systematic literature review of built environment effects on physical activity and active transport – an update and new findings on health equity. Int J Behav Nutr Phys Act (2017) 14:158 10.1186/s12966-017-0613-9 29145884 PMC5693449

[8] Berkowitz SA Berkowitz TSZ Meigs JB Wexler DJ. Trends in food insecurity for adults with cardiometabolic disease in the United States: 2005–2012. PLoS One (2017) 12(6):e0179172. 10.1371/journal.pone.0179172 28591225 PMC5462405

[9] Wang EA McGinnis KA Goulet J Bryant K Gibert C Leaf DA. Food insecurity and health: data from the Veterans Aging Cohort Study. Public Health Rep (2015) 130(3):261–8. 10.1177/003335491513000313 25931630 PMC4388224

[10] Office of Disease Prevention and Health Promotion. Healthy People 2020 Topics and Objectives: Access to Health Services. (2018). Available from: https://www.healthypeople.gov/2020/topics-objectives/topic/Access-to-Health-Services

[11] Israel BA Schulz AJ Parker EA Becker AB Allen AJ Guzman JR Critical Issues in Developing and Following CBPR Principles. In: Minkler M Wallerstein N, editors. CBPR for Health. San Francisco, CA: Jossey Bass (2003). 47–62 p.

[12] Israel B Coombe C Cheezum R Schulz AJ McGranaghan RJ Lichtenstein R. Community-based participatory research: a capacity-building approach for policy advocacy aimed at eliminating health disparities. Am J Public Health (2010) 100(11):2094–102. 10.2105/AJPH.2009.170506 20864728 PMC2951933

[13] Minkler M. Linking science and policy through community-based participatory research to study and address health disparities. Am J Public Health (2010) 100(Suppl):S81–7. 10.2105/AJPH.2009.165720 20147694 PMC2837446

[14] Wallerstein NB Duran B. Using community-based participatory research to address health disparities. Health Promot Pract (2006) 7(3):312–23. 10.1177/1524839906289376 16760238

[15] Lewis D Yerby L Tucker M Foster PP Hamilton KC Fifolt MM Bringing community and academic scholars together to facilitate and conduct authentic community based participatory research: project UNITED. Int J Environ Res Public Health (2016) 13(1):35 10.3390/ijerph13010035 PMC4730426 26703675

[16] Rubin CL Allukian N Wang X Ghosh S Huang C-C Wang J “We make the path by walking it”: building an academic community partnership with Boston Chinatown. Prog Community Health Partnersh (2014) 8(3):353–63. 10.1353/cpr.2014.0046 25435562 PMC4519822

[17] Israel B Parker E Rowe Z Community-based participatory research: lessons learned from the centers for children’s environmental health and disease prevention research. Environ Health Perspect (2005) 113(10):1463–71. 10.1289/ehp.7675 16203263 PMC1281296

[18] Resnik DB Kennedy CE. Balancing scientific and community interests in community-based participatory research. Account Res (2010) 17(4):198–210. 10.1080/08989621.2010.493095 20597018 PMC3074227

[19] Wallerstein N. Power between evaluator and community: research relationships within New Mexico’s healthier communities. Soc Sci Med (1999) 49(1):39–53. 10.1016/S0277-9536(99)00073-8 10414839

[20] Coats JV Stafford JD Thompson VS Javois BJ Goodman MS Increasing research literacy: The Community Research Fellows Training Program. J Empirical Res Hum Res Ethn (2014) 10(1):3–12. 10.1177/1556264614561959 PMC4605821 25742661

[21] D’Agostino McGowan L Stafford JD Thompson VL Johnson-Javois B Goodman MS. Quantitative evaluation of the Community Research Fellows Training Program. Front Public Health (2015) 3:179. 10.3389/fpubh.2015.00179 26236703 PMC4504145

[22] Goodman MS Thompson VS, editors. Public Health Research Methods for Partnerships and Practice. New York: Routledge (2018).

[23] Goodman MS Dias JJ Stafford JD. Increasing research literacy in minority communities: CARES Fellows Training Program. J Empir Res Hum Res Ethics (2010) 5(4):33–41. 10.1525/jer.2010.5.4.33 21133785 PMC3177406

[24] Goodman MS Si X Stafford JD Obasohan A Mchunguzi C. Quantitative assessment of participant knowledge and evaluation of participant satisfaction in the CARES Training Program. Prog Community Health Partnersh (2012) 6(3):361–8. 10.1353/cpr.2012.0051 22982849 PMC4849880

