# Supplementary material for: Increasing Research Capacity in Underserved Communities: Formative and Summative Evaluation of the Mississippi Community Research Fellows Training Program (Cohort 1)
Source: Front Public Health. 2018 Feb 9;6:21. doi: 10.3389/fpubh.2018.00021 (PMC5811515; doi:10.3389/fpubh.2018.00021)
Supplement: Supplementary file 1 [file image_1.PDF]

Supplementary Material

Increasing Research Capacity in Underserved Communities: Formative and Summative Evaluation of the Mississippi Community Research Fellows Training Program (Cohort One)

Danielle Fastring, PhD, MPH[1*], Susan Mayfield Johnson, PhD, MPH[1], Tanya Funchess, DHA, MPH, MSM[2], Candice Green, MPH[2], Victoria Walker, MPH[3], Georgette, Powell, MPH[2]

[1]Department of Public Health, University of Southern Mississippi, Hattiesburg, MS, USA

[2] Office of Health Disparity Elimination, Mississippi State Department of Health, Jackson, MS USA

[3]Office of Policy and Evaluation, Mississippi State Department of Health, Jackson, MS, USA

*** Correspondence:**
Danielle Fastring, PhD, MPH
Danielle.fastring@usm.edu

Supplementary Figure 1. Logic Model for MSCRFT Program

**Inputs**	Steering Committee / CAB	MSCRFT Faculty	Financial Support from MSDH	MSDH Staff
**Activities**	Adapted Curriculum to Ensure Cultural Competency	Delivered Culturally Competent Curriculum in 15 Weekly Sessions	Managed Faculty. Managed Data Collection, Day to Day Operations	
**Outputs**	Culturally Competent curriculum	Participant Training Portal: Faculty Contact Information, Participant Contact Information, Accessible Training Materials, Participant Tool Kit		Database for Baseline, Midpoint, and Final Assessment
**Short-Term Outcomes**	Increase Participant Knowledge of Key Concepts	Increase Participant Skills in Community Assessment (Park Audit, Grocery Store Audit, Data-Driven Problem Identification)		Process Evaluation
**Intermediate Outcomes**	Participants Become More Involved in Community Events	Participants Become More Empowered to Advocate for Community Needs	Participants Conduct Community Assessments in Their Neighborhoods	Outcome Evaluation
**Long-Term Outcomes**	Increase Capacity for CBPR Between Academia, Public Health Workers, Community-Based Entities, and CHWs Serving Mississippi	Increase Role of Racial/Ethnic Minorities and Other Underserved Populations in CBPR		Impact Evaluation
