# Supplementary material for: Increasing Research Capacity in Underserved Communities: Formative and Summative Evaluation of the Mississippi Community Research Fellows Training Program (Cohort 1)
Source: Front Public Health. 2018 Feb 9;6:21. doi: 10.3389/fpubh.2018.00021 (PMC5811515; doi:10.3389/fpubh.2018.00021)
Supplement: Supplementary file 2 [file table_1.docx]

Supplementary Material

Increasing Research Capacity in Underserved Communities: Formative and Summative Evaluation of the Mississippi Community Research Fellows Training Program (Cohort One)

Danielle Fastring, PhD, MPH^1*^, Susan Mayfield Johnson, PhD, MPH^1^, Tanya Funchess, DHA, MPH, MSM^2^, Candice Green, MPH^2^, Victoria Walker, MPH^3^, Georgette, Powell, MPH^2^

^1^Department of Public Health, University of Southern Mississippi, Hattiesburg, MS, USA

^2^ Office of Health Disparity Elimination, Mississippi State Department of Health, Jackson, MS USA

^3^Office of Policy and Evaluation, Mississippi State Department of Health, Jackson, MS, USA

*** Correspondence:**Danielle Fastring, PhD, MPH
Danielle.fastring@usm.edu

**
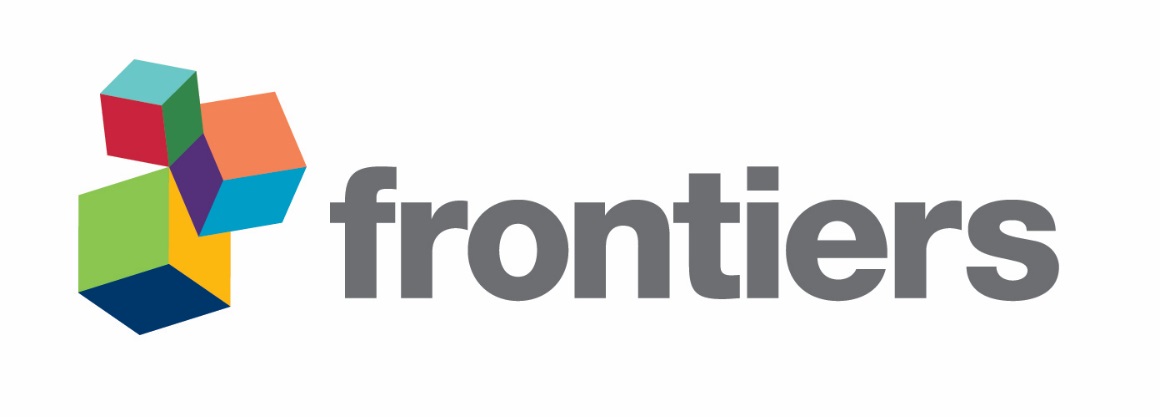
**

**Supplementary Table 1.** Session Topics and Learning Objectives

|  | **TOPICS** | **LEARNING OBJECTIVES** |
| --- | --- | --- |
| Session 1 | Evidence Based Public Health | - Define evidence based public health - Identify public resources available for public health |
| Session 2 | Research Methods  Data | - Define Research - Describe the steps of the research process - Identify and explain research methodology - Identify appropriate research methods and techniques - Define data - Compare and contrast quantitative and qualitative data - Compare and contrast primary data and secondary data - Describe strengths of mixed methods approaches |
| Session 3 | Health Disparities | - Define health disparities. - Identify major health disparities in Mississippi including those by gender, race/ethnicity, geographic location, and socioeconomic status - Discuss the social determinants of health - Describe public health strategies and interventions for reducing health disparities |
| Session 4 | Cultural Competency | - Define culture and cultural competency - Describe the need for culturally competent research and practice based on a historical perspective - Identify skills associated with culturally competent practices |
| Session 5 | Family Health History  Health Literacy | - Understand importance of collecting and maintaining a family health history - Understand the role of family health history in healthcare - Complete a family history chart - Define health literacy - Understand the limited literacy perspective - Describe the association between literacy and health - Describe health literacy on a national scale |
| Session 6 | Introduction to Epidemiology | - Define epidemiology - Identify major contributions of epidemiology   • Identify frameworks for understanding disease  processes  • Compare and contrast observational studies vs.  clinical trials |
| Session 7 | Community Health | • Define community health  • Identify contributing factors that impact the health  of a community.  • Describe community health activities  • Discuss principals for community based prevention.  • Assess the need for a community program |
| Session 8 | Quantitative Methods | - Identify strengths and weaknesses of quantitative methods - Describe strengths of mixed methods approaches - Describe stages of questionnaire design - Identify sampling methods - Understand usefulness of statistics in health research - Understand p-values and odds ratios |
| Session 9 | Community-Based Participatory Research | - Describe history and principles of CBPR - Critically evaluate fellows’ position within their community (ies) and their potential roles within CBPR projects - Describe methods to ensure that CBPR benefits all partners |
| Session 10 | Qualitative Methods | - Define basic principles of qualitative research methods - Describe the strengths and weaknesses of qualitative methods - Discuss different types of qualitative approaches - Discern when a qualitative research design is desirable |
| Session 11 | Photo Voice  Health Policy | - Define and discuss concepts of Photovoice - Understand focus groups and Photovoice qualitative research methods - Discuss the usage of Photovoice in public health - Define health policy and health services research - Identify and develop relevant well framed health policy research questions - Describe public use and other common data sources for health policy research |
| Session 12 | Program Evaluation | - Develop SMART objectives for programs and projects - Compare and contrast goals and objectives - Identify culturally competent evaluation approaches - Understand the importance of evaluation |
| Session 13 | Research Ethics  Human Subjects Certification | - Define research ethics and bioethics - Compare and contrast clinical ethics vs research ethics - Identify examples of unethical practices in research - Understand ethical theories and professional ethical duties - Participants will understand the importance of human subjects certification. |
| Session 14 | Clinical Trials | - Understand clinical trials research - Describe the role of clinical trials research in advancing medical practice - Discuss the impact of minority participation in clinical trials research |
| Session 15 | Grant Writing | - Understand grant guidelines and requirements - Understand the power of collaboration for grant writing - Develop SMART goals and specific Aims - Understand components of a good grant proposal |
